# Targeting R-loop-associated ATR response in myelodysplastic syndrome

**DOI:** 10.18632/oncotarget.26851

**Published:** 2019-04-05

**Authors:** Hai Dang Nguyen, Lee Zou, Timothy A. Graubert

**Affiliations:** Lee Zou: Massachusetts General Hospital Cancer Center, Harvard Medical School, Charlestown, MA, USA; Department of Pathology, Massachusetts General Hospital, Harvard Medical School, Boston, MA, USA; Timothy A. Graubert: Massachusetts General Hospital Cancer Center, Harvard Medical School, Charlestown, MA, USA

**Keywords:** myelodysplastic syndrome, splicing factor mutations, U2AF1, ATR, R-loop

Recent genomic characterization of cancers has revealed recurrent somatic mutations in genes encoding RNA splicing factors. As many as 119 splicing factors were implicated in a recent pan-cancer analysis [1], highlighting a strong link between RNA splicing regulation and cancer pathogenesis. Somatic heterozygous hotspot mutations in a specific component of the core spliceosome U2 complex (*SF3B1*) and two splicing regulators bound to 3’ splice sites (*U2AF1*) or exonic splicing enhancers (*SRSF2*) are particularly prevalent and mutually exclusive [2]. The frequency of mutations in these three factors is highest in hematological malignancies, such as myelodysplastic syndrome (MDS) and acute myeloid leukemia, and is significant but lower in solid tumors [2]. Intriguingly, the splicing alterations induced by mutant splicing factors are largely non-overlapping, raising the possibility that the functional consequences of these mutations may be mediated by additional mechanisms beyond perturbation of splicing.

**Figure 1:**
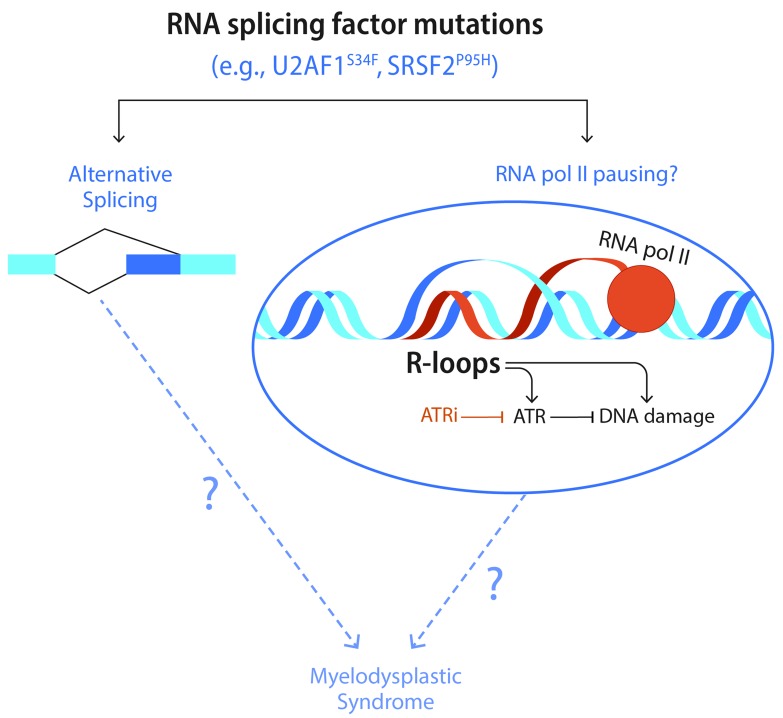
Spliceosome mutations confer an R loop-dependent vulnerability MDS-associated spliceosome mutants induce alternative RNA splicing, which may contribute to MDS pathogenesis. The spliceosome mutants also induce R-loops, possibly owing to the stalling of RNA polymerase II. Whether and how R-loops contribute to MDS pathogenesis is still unknown. Nonetheless, R-loops trigger an ATR response and render cells dependent on ATR for survival.

Growing evidence suggests that defective RNA processing can induce accumulation of R-loops, transcription intermediates consisting of RNA:DNA hybrids and displaced single-stranded DNA (ssDNA) [3]. We previously reported that expression of an MDS-associated U2AF1(S34F) mutant induces R-loop accumulation [4]. Interestingly, we further showed that suppression of R-loops by expressing RNaseH1, an enzyme that specifically cleaves the RNA in RNA:DNA hybrids, did not rescue the known alternative splicing events induced by the U2AF1 mutant [5]. In agreement with our findings, a genome-wide analysis performed by Chen et al. suggested that R-loops accumulate in cells expressing the MDS-associated U2AF1 or SRSF2 mutants, but these R-loops are not enriched at the alternatively-spliced junctions [6]. These studies suggest that RNA splicing perturbation and R-loop accumulation may be two independent processes affected by the splicing factor mutations in MDS (Figure 1).

It is not yet known how the increase of R-loops impacts the cancer genome. R-loops are known to interfere with DNA replication and induce replication stress [7]. Furthermore, R-loops also play important roles in a number of normal cellular processes, such as transcription, telomere maintenance, and chromosome segregation [3]. Thus, in addition to inducing replication stress, the aberrant R-loops in cells expressing spliceosome mutants may affect a spectrum of cellular processes. It is possible that only a subset of the aberrant R-loops in MDS cells becomes an intrinsic source of replication stress. Regardless of where aberrant R-loops induce replication stress in the genome, we observed that the ATR kinase is activated in an R-loop dependent manner in cells expressing MDS-associated U2AF1 mutant alleles [5]. Importantly, the ATR response to aberrant R-loop accumulation is critical for cell survival since ATR inhibition selectively induced DNA damage and reduced viability in cells expressing spliceosome mutants. Although we demonstrated that ATR is activated by the aberrant R-loops in cells expressing spliceosome mutants, the underlying mechanism is not fully understood. ATR could directly associate with R-loops through ssDNA coated by Replication Protein A (RPA). Alternatively, R-loops could impede DNA replication forks, thereby activating ATR through fork stalling. Since the ATR kinase is a master guardian against different sources of genomic instability, ATR may phosphorylate specific substrates to resolve aberrant R-loops. In future studies, it will be important to determine how aberrant R-loop accumulation induces ATR activity and how ATR functions to resolve R-loops and prevent genomic instability.

Emerging evidence has also suggested that the normal function of ATR may be altered in cells with spliceosome mutations. First, alternatively spliced ATR transcripts were found in AML patients harboring *U2AF1* mutations [8]. An independent study also found alternatively spliced ATR transcripts specifically enriched in the granulomonocytic lineage derived from CD34+ cells transduced with mutant U2AF1 [9]. Interestingly, a murine model expressing an U2AF1 mutant also exhibited increased genomic instability in monocytic cells [10]. How these features impact R-loop regulation and other ATR cellular functions in MDS is not yet known.

Based on these pre-clinical findings, we recently initiated a single-arm phase Ib trial to assess safety and seek preliminary evidence of efficacy for the ATR inhibitor, AZD6738, in patients with MDS and chronic myelomonocytic leukemia who have failed first line therapy (NCT03770429). We will test the hypothesis that patients with spliceosome mutations are more likely to respond to ATR inhibition and will explore potential genetic and epigenetic mechanisms of resistance. Future goals will be to identify rational combination strategies incorporating ATR inhibition in MDS treatment. Ongoing basic and translational research should provide mechanistic insights into R-loops and the ATR response, as well as additional targetable vulnerabilities and biomarkers that can be used for patient selection and to monitor responses. Furthermore, it will be important to address whether aberrant R-loop accumulation is common in other cancers harboring spliceosome mutations. If aberrant R-loop accumulation is a common vulnerability in cancers, ATR inhibitors may have a broad therapeutic potential in cancer therapy.
